# Enhanced functional connectivity involving the ventromedial hypothalamus following methamphetamine exposure

**DOI:** 10.3389/fnins.2015.00326

**Published:** 2015-09-23

**Authors:** Damian G. Zuloaga, Ovidiu D. Iancu, Sydney Weber, Desiree Etzel, Tessa Marzulla, Blair Stewart, Charles N. Allen, Jacob Raber

**Affiliations:** ^1^Department of Behavioral Neuroscience, Oregon Health & Science University PortlandPortland, OR, USA; ^2^Department of Psychology, University at AlbanyAlbany, NY, USA; ^3^Oregon Institute of Occupational Health Sciences, Oregon Health & Science University PortlandPortland, OR, USA; ^4^Department of Neurology, Oregon Health & Science University PortlandPortland, OR, USA; ^5^Department of Radiation Medicine, Oregon Health & Science University PortlandPortland, OR, USA; ^6^Division of Neuroscience, ONPRC, Oregon Health & Science University PortlandPortland, OR, USA

**Keywords:** methamphetamine, light phase, dark phase, activation, functional connectivity

## Abstract

Methamphetamine (MA) consumption causes disruption of many biological rhythms including the sleep-wake cycle. This circadian effect is seen shortly following MA exposure and later in life following developmental MA exposure. MA phase shifts, entrains the circadian clock and can also alter the entraining effect of light by currently unknown mechanisms. We analyzed and compared immunoreactivity of the immediate early gene c-Fos, a marker of neuronal activity, to assess neuronal activation 2 h following MA exposure in the light and dark phases. We used network analyses of correlation patterns derived from global brain immunoreactivity patterns of c-Fos, to infer functional connectivity between brain regions. There were five distinct patterns of neuronal activation. In several brain areas, neuronal activation following exposure to MA was stronger in the light than the dark phase, highlighting the importance of considering circadian periods of increased effects of MA in defining experimental conditions and understanding the mechanisms underlying detrimental effects of MA exposure to brain function. Functional connectivity between the ventromedial hypothalamus (VMH) and other brain areas, including the paraventricular nucleus of the hypothalamus and basolateral and medial amygdala, was enhanced following MA exposure, suggesting a role for the VMH in the effects of MA on the brain.

## Introduction

The abuse of the psychostimulant methamphetamine (MA) causes great physiological (Wolkoff, 1997), social (Sommers et al., 2006), and financial damage (Dobkin and Nicosia, 2009). It is estimated to cost the United States approximately $23.4 billion annually (Dobkin and Nicosia, 2009). In the United States, MA was abused by approximately 439,000 people in 2011, 133,000 of which were new users (SAMHSA 2012).

MA consumption by humans is characterized by disruption of many biological rhythms including the sleep-wake cycle (Hasler et al., 2012). This circadian effect is not only seen relatively shortly following MA exposure (Honma et al., 1986, 1991; Uchihashi et al., 1994; Masubuchi et al., 2001; Olsen et al., 2013), but also later in life following developmental MA exposure (Eastwood et al., 2012). MA phase shifts, entrains the circadian clock, and can also alter the entraining effect of light by currently unknown mechanisms (Honma et al., 1986). The circadian rhythms of mice or rats made arrhythmic by lesions of the suprachiasmatic nucleus (SCN) can be regenerated by consumption of MA dissolved in the drinking water (Honma et al., 1986; Tataroglu et al., 2006; Mohawk et al., 2009). These reconstituted rhythms are robust and show periods significantly longer than the wild-type rhythms. These observations led to the hypothesis that a separate MethAmphetamine-Sensitive Circadian Oscillator (MASCO) exists in the brain separate from the SCN (Tataroglu et al., 2006). The MASCO is not composed of the canonical clock genes since animals made arrhythmic by knocking out these clock genes continue to demonstrate robust MA-induced rhythms (Masubuchi et al., 2001; Mohawk et al., 2009). To date, the location of this oscillator and its molecular make up remain completely unknown (Tataroglu et al., 2006). Although the SCN might play a role in MASCO, it does not seem critical as the effects of MA on circadian system are also seen in animals in which the SCN is lesioned (Tataroglu et al., 2006).

The ventromedial hypothalamus (VMH) might play a role in the circadian effects of MA. The VMH is involved in synchronization of rhythms to periodic feeding but, unlike the SCN, does not contain a self-sustained oscillator associated with food (Inouye, 1983). The VMH is also important for circadian rhythms in serum insulin, glucose, and triglycerides (Egawa et al., 1993). Disturbed circadian feeding rhythmicity and inhibition of the glucose preference reversal are observed in rats with VMH lesions, a functional state similar to that seen following chronic MA exposure (Kraeuchi et al., 1985; Mitsushima et al., 1994). The VMH might be involved in other circadian rhythms as well. Implantation of a hydro-polymer gel into the VMH to alter its neural network, likely involving neural output via a dorsal route, showed attenuation of circadian activity levels in blind female rats and of circadian changes in serum melatonin levels (Mitsushima et al., 1994).

The adrenocorticotropic hormone (ACTH) secretagogue arginine vasopressin (AVP) (Dornhorst et al., 1981; Antoni, 1986) plays a role in circadian effects. Intrahypothalamic injection of an AVP V1 receptor blocker reveals the diurnal stimulatory input of AVP to Hypothalamus-Pituitary-Adrenal (HPA) axis activation (Kalsbeek et al., 1996). Release of AVP in the paraventricular nucleus of the hypothalamus (PVN) is also seen following emotional stress (Wotjak et al., 1996), local inflammation (Turnbull and Rivier, 1996), and inflammatory cytokines (Raber et al., 1998). The PVN showed profound activation by MA and an increase in the number of c-Fos/AVP dual-labeled cells during the light phase (Zuloaga et al., 2014). It is unclear whether these effects are restricted to the light phase or seen in the dark phase as well.

In most studies, there is a bias to study a particular brain region or network that is pertinent to a specific behavioral or cognitive change seen following MA exposure or other drugs of abuse (Abekawa et al., 1996; Broening et al., 1997; Chang et al., 2002, 2004, 2009; Brady et al., 2003; Koob and Volkov, 2010). In the current study, we analyzed and compared immunoreactivity of the immediate early gene c-Fos, a marker of neuronal activity (French and Pavlidis, 2011), to assess neuronal activation 2 h following MA exposure in the light and dark phases. We also compared co-localization of c-Fos with AVP in the PVN in the light and dark phases. Finally, we used network analyses of functional connectivity based on global brain immunoreactivity patterns of c-Fos, to infer functional connectivity between brain regions (Schiltz et al., 2007). We hypothesize that a brain region showing a neuronal activation pattern in the light and dark phases that resembles that of the SCN and shows a functional neuronal connectivity that is enhanced following MA exposure might suggest its involvement in MASCO.

## Materials and methods

### Animals

Forty male C57BL6/J mice, 28–42 days old, were purchased from JAX Laboratories (Bar Harbor, Maine) and maintained on a 12/12 light/dark cycle with lights on at 5:00 am (Zeitgeber Time 0, ZT0). Mice were singly housed for 4 days prior to experiments and were between 50 and 70 days old at the time of testing. Rodent chow (PicoLab Rodent Diet 20, #5053; PMI Nutrition International, St. Louis, MO) and water were available *ad libitum*.

### Animal approval

All procedures complied with the National Institutes of Health Guide for the Care and Use of Laboratory Animals and with IACUC approval at Oregon Health & Sciences University.

### MA treatment and c-Fos immunohistochemistry

The mice were injected with (*d*)-MA hydrochloride dissolved in saline (1 mg/kg, ip.) or saline alone between 3 and 4 h after lights on (ZT3–4) or 3–4 h after lights off (ZT15–16). Two hours later, the mice were intracardially perfused with 20 ml of phosphate buffered saline (PBS), followed by 40 ml of a 4% paraformaldehyde solution. Brains were removed, stored in 4% paraformaldehyde overnight, and then transferred to a 30% sucrose solution. The 2 h time point was chosen based on our previous study that demonstrated extensive c-Fos induction in the mouse brain following administration of the same dose of MA (Zuloaga et al., 2014). The mice were injected and perfused in separate cohorts so that all mice in a cohort were perfused within 82 min. Fixed brains were coronally sectioned at 40 μm into three series using a cryostat (Microm HM505E, MICROM international GmbH, Walldorf, Germany) and processed for immunohistochemical detection of c-Fos. Sections were rinsed in PBS, incubated in 1% hydrogen peroxide and 0.3% Triton-X in PBS (PBS-TX) for 10 min, again rinsed in PBS, then incubated in 10% normal goat serum (NGS) in PBS-TX for 1 h. After rinsing in PBS, sections were incubated in primary antisera (c-Fos rabbit polyclonal: 1:5000, Santa Cruz Biotechnology, sc52) in 4% NGS and PBS-TX overnight at room temperature. Sections were then rinsed in PBS and incubated for 1 h in biotinylated goat-anti rabbit antibody in PBS-TX (1:500, Vector Laboratories, Burlingame, CA), followed by rinses in PBS and a 1 h incubation in avidin-biotin peroxidase complex (ABC Elite kit, Vector Laboratories, Burlingame, CA). Following rinses in Tris buffered saline (TBS), sections were developed for visualization of c-Fos positive cells in a hydrogen peroxide/diaminobenzidine/TBS solution for 10 min, after which sections were rinsed in PBS and immediately mounted on slides. The following day, sections were dehydrated in ethanol, defatted in xylene, and coverslipped with Permount (Sigma Chemical Co., St. Louis MO).

### Dual label immunohistochemistry

To determine the co-localization of c-Fos and AVP immunoreactivity in the mouse brain, we performed dual label immunohistochemistry. Free-floating sections were rinsed with PBS 3 times, then blocked with 4% donkey serum in PBS-TX for 90 min. Sections were then incubated in anti-c-Fos (1:250, goat, Santa Cruz Biotechnology, sc52-G, Billerica, MA) overnight. Sections were then incubated in 1:200 donkey anti-goat Dylight 594 (Abcam, Cambridge, MA, USA) for 3 h at room temperature. Sections were then rinsed in PBS 4 times (20 min each rinse) after which the same protocol was repeated using AVP (1:500, rabbit, gift from Dr. Paul Plotsky) as primary antibody and 1:200 donkey anti-rabbit Alexa 488 (Life Technologies) as the secondary antibody. Sections were slide mounted and coverslipped with antifade reagent to preserve fluorescent signal (Vectashield with DAPI, Vector), light protected, and stored at 4°C.

### Microscopy

Quantification of c-Fos positive cells was performed using an Olympus IX81 microscope (Olympus, Center Valley, PA, USA) equipped with Slidebook software (Intelligent Imaging Innovations, Inc., Denver, CO, USA). Images of discrete brain regions were captured bilaterally within two sections using a 10 × objective. These regions were mainly selected based on our previous study showing robust neuronal activation in these areas following MA exposure during the light phase (Zuloaga et al., 2014). Regions were identified using the mouse brain atlas (Franklin and Paxinos, 1997). c-Fos immunoreactive cells (identified by black nuclear label) were quantified bilaterally within fixed area frames; infralimbic cortex (ILC; Bregma 1.94–1.78; box, 580 × 520 μm), SCN (Bregma -0.82 to -0.94; 740 × 420 μm), PVN (Bregma -0.82 to -0.94; box, 275 × 450 μm), paraventricular thalamic nucleus (PVT; Bregma −0.82 to −0.94; box, 790 × 410 μm each), bed nucleus of the stria terminalis (BNST; Bregma 0.02 to −0.10; box, 335 × 620 μm), central amygdala (CEA; Bregma −1.34 to −1.46; circle, 575 μm diameter), basolateral amygdala (BLA; Bregma −1.34 to −1.46; circle, 450 μm diameter), medial amygdala (MEA; Bregma 0.02 to −0.10; box, 460 × 400 μm), cingulate cortex area 2 (CIN; Bregma −1.34 to −1.46; box, 428.28 × 394.74 μm), nucleus accumbens core (NACc; Bregma 1.1–0.98; circle, 652.74 μm in diameter), nucleus accumbens shell (NACs; Bregma 1.1–0.98, box, 300 × 350 μm), arcuate nucleus (ARC; Bregma −1.46 to −1.58; right triangle, 420 × 380 μm), VMH (Bregma −1.46 to −1.58; circle, 380 μm diameter), dorsomedial hypothalamus (DMH; Bregma −1.46 to −1.58; 440 × 550 μm), dentate gyrus (DG; Bregma -0.82 to -0.94; box, 850 × 420 μm), CA1 region of the hippocampus (CA1; Bregma −0.82 to −0.94; box, 850 × 420 μm), and CA3 region of the hippocampus (CA3; Bregma −0.82 to −0.94; box, 850 × 420 μm).

For quantification of c-Fos/AVP dual labeling, confocal images of the PVN were captured bilaterally at 20 × in two sections using the same Olympus IX81 confocal microscope. c-Fos/AVP dual-labeled cells were quantified within two regions of the PVN, the lateral magnocellular (circle, 135 μm diameter) and the medial parvocellular regions (circle, 135 μm diameter). Cells were considered co-localized when they expressed both red (c-Fos) and green (AVP) fluorescence. For AVP/c-fos co-expressing cells AVP was found within the extra-nuclear area, while c-fos was localized to the nucleus, resulting in a green cell body that surrounds a red nucleus. The number of c-Fos/AVP expressing cells in the magnocellular and parvocellular regions were counted within each brain and the data are expressed as the mean number of c-Fos/AVP positive cells per experimental group.

### Functional connectivity analysis

Using the c-Fos immunohistochemical data (single labeling with biotinylated secondary antibody), we inferred a measure of functional connectivity between brain regions, following the general methodology introduced by Schiltz et al. (2007). We based our analysis on the average number of activated cells in each brain. These values were correlated across samples, and significant correlations were considered evidence of functional connectivity or coupling between the respective brain regions. These values were correlated across samples, and significant correlations were considered evidence of functional connectivity or coupling between the respective brain regions. As there was no interaction between time of day and MA treatment, we collapsed the data of the light and dark phases for the connectivity analysis. Combining the data from the light and dark phases also increased the power of the analysis. To examine the effects of MA exposure on functional connectivity, we quantified the functional connectivity between the regions for animals exposed to MA vs. saline controls. We correlated the relative activation levels of each brain region across individuals/samples and we considered these measures to reflect the levels of functional coupling between distinct brain regions. For each region, the sum of absolute levels of correlations with all other regions was denoted as the *connectivity*. To determine whether these connectivity values could arise by chance, we employed a permutation procedure. We randomized the order of the samples and recomputed the correlations, with the expectation that correlation values and consequently connectivity values that arise from chance randomized sample orders will be significantly lower than the values inferred from the unperturbed data. We divided the data in two sets of MA exposed and saline exposed animals, respectively, and we constructed networks from these two groups. In these separate networks, we observed differences in connectivity values of brain regions. To evaluate whether these changes reach statistical significance, we employed a bootstrapping procedure utilized in comparing MRI-derived functional connectivity networks (Hosseini and Gat, 2012), as well as in comparing gene co-expression networks (Gill et al., 2014). We combined the MA exposed and saline exposed data and we separated the combined data in two sets (*n* = 10,000 random resamples); for each of these resamples we constructed two different networks and we recorded the difference in connectivity. The difference between the MA exposed and saline exposed networks was compared to the distribution of differences arising from mixed-sample networks.

### Statistical analyses

All data are reported as means ± standard error of the mean (SEM). Data were analyzed using GraphPad Prism v.4 and SPSS v.16.0 software (Chicago, IL). Immunohistochemical data were analyzed using Two-Way ANOVA with time and treatment as factors. For the functional connectivity analyses, R software was used. Bonferroni corrected post hoc comparisons were performed when statistically appropriate. Using a bootstrapping procedure, we derived statistical significance for changes in the patterns of connectivity.

## Results

### c-Fos quantification

For each brain area, we analyzed whether there were significant effects of treatment, time of day, and a treatment × time interaction. In brain areas in which there was a time × treatment interaction, we also analyzed the effects of the two treatments (i.e., MA and saline) separately. Based on the statistical analysis, there were five distinct patterns of neuronal activation: (1) brain areas that showed an effect of treatment, but no effect of time or time × treatment interaction: (2) brain areas that showed an effect of time, but no effect of treatment or time × treatment interaction; (3) brain areas that showed an effect of treatment and a treatment × time interaction; (4) brain areas that showed effects of treatment and time but no time × treatment interaction; and (5) brain areas that show no effects of treatment or time and no time × treatment interaction. Each pattern is described separately below. Please see also Table 1 for an overview of the statistical effects.

**Table 1:** **Patterns of neuronal activity**.

**Pattern**	**Treatment effect**	**Time effect**	**Treatment × Time interaction**	**Brain areas**
1	Yes	No	No	ILC, CEA, BNST, CA1, CIN, NACc
2	No	Yes	No	SCN, VMH, DMH
3	Yes	No, only yes in PVN	Yes	PVN, PVT, ARC
4	Yes	Yes	No	CA3, NACs
5	No	No	No	MEA, BLA, DG

### Pattern 1. brain areas that showed only an effect of treatment

**Figure 1:**
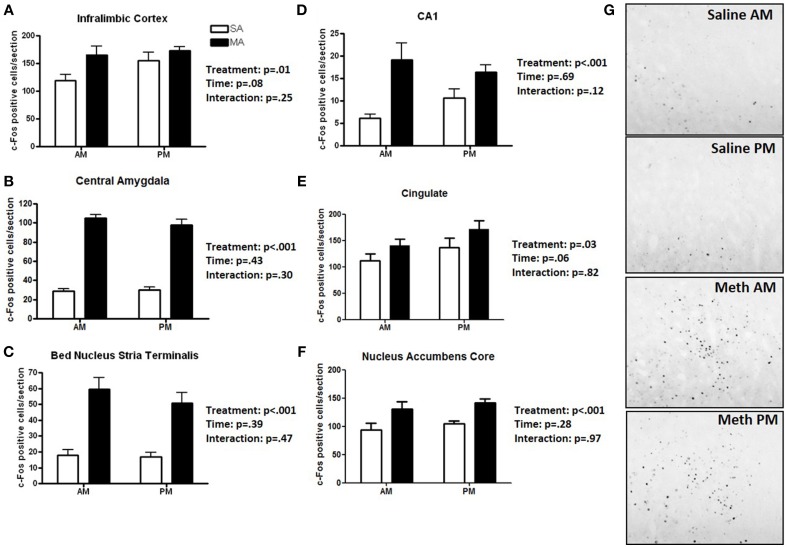
**Brain areas that showed only an effect of treatment**. Following MA exposure, there was an increase in c-Fos immuno-positive cells in the ILC **(A)**, CEA **(B)**, BNST **(C)**, CA1 **(D)**, CIN **(E)**, and NACc **(F)**. Representative images for neuronal activation in the CEA following saline or MA exposure in the day (AM) and night (PM) are shown in **(G)**. *n* = 10 mice/treatment/time period.

Six brain regions showed an overall increase in c-Fos positive cells following MA treatment in the light and dark phases (Figure 1). There was a significant main effect of MA for the number of c-Fos immunopositive cells in the ILC [*F*_(1, 36)_ = 14.19, *p* = 0.014; Figure 1A), CEA [*F*_(1, 36)_ = 90.17, *p* < 0.001; Figure 1B), BNST [*F*_(1, 36)_ = 90.17, *p* < 0.001; Figure 1C), CA1 [*F*_(1, 36)_ = 16.61, *p* < 0.001; Figure 1D], CIN [*F*_(1, 36)_ = 4.85, *p* < 0.05; Figure 1E], NACc [*F*_(1, 36)_ = 23.28, *p* < 0.001; Figure 1F]. Representative images for neuronal activation in the CEA following saline or MA exposure in the day (AM) and night (PM) are shown in Figure 1G.

### Pattern 2. brain areas that showed only an effect of time

**Figure 2:**
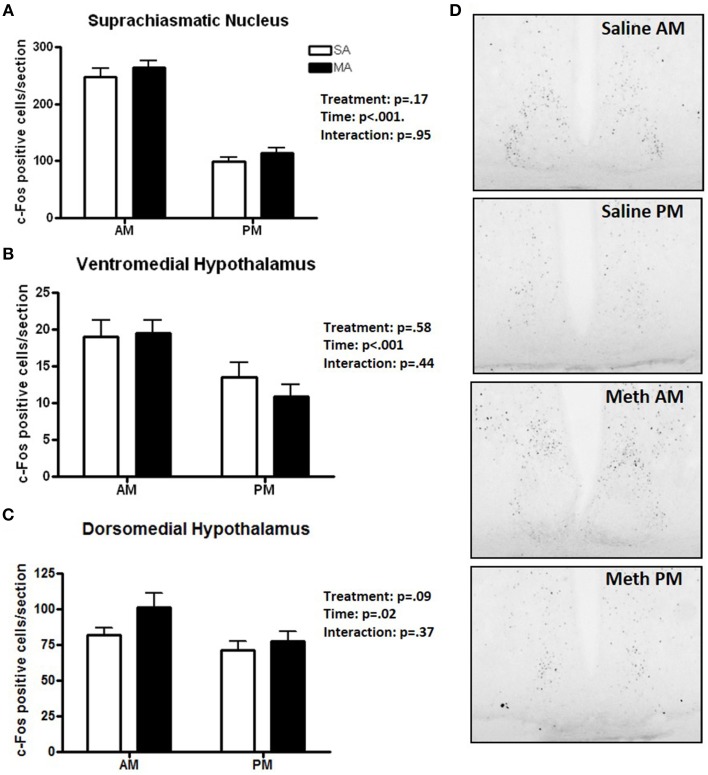
**Brain areas that showed only an effect of time**. The number of c-Fos immuno-positive cells is higher in the day than night in the SCN **(A)**, VMH **(B)**, and DMH **(C)**. Representative images for neuronal activation in the SCN following saline or MA exposure in the day and night are shown in **(D)**. *n* = 10 mice/treatment/time period.

Three brain regions showed significantly more c-Fos positive cells in the light than dark period. A significant main effect of time, but no significant effects of MA, was found for the number of c-Fos cells in the SCN [*F*_(1, 36)_ = 185.78, *p* < 0.001; Figure 2A], VMH [*F*_(1, 36)_ = 12.92, *p* < 0.001; Figure 2B], and DMH [*F*_(1, 36)_ = 5.48, *p* = 0.02; Figure 2C]. Representative images for neuronal activation in the SCN following saline or MA exposure in the day and night phases are shown in Figure 2D. There were trends toward an effect of time in the ILC (*p* = 0.08, Figure 1A) and CIN (*p* = 0.06, Figure 1E) but that did not reach statistical significance.

### Pattern 3. brain areas that showed effects of treatment and a treatment × time interaction

**Figure 3:**
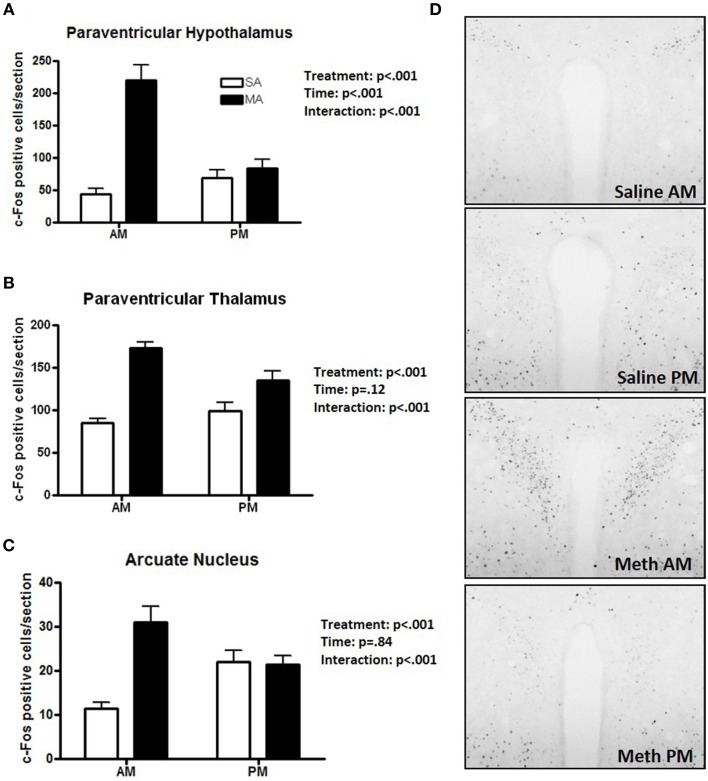
**Brain areas that showed effects of treatment and a treatment × time interaction**. The increase in the number of c-Fos positive cells following MA exposure is greater in the day than in the night in the PVN **(A)**, PVT **(B)**, and ARC **(C)**. In these three brain regions there was a MA × time interaction. In the dark phase, the number of c-Fos positive cells was only higher following MA than saline exposure in the PVT (*p* = 0.028) but not in the PVN or ARC. In the PVN (*p* < 0.0001), PVT (*p* = 0.01), and ARC (*p* = 0.03) neuronal activation following MA exposure was significantly greater in the light than dark phase. In contrast, in the ARC, neuronal activation following MA exposure was significantly greater in the dark than the light phase (*p* < 0.01). In the PVN (*p* = 0.12) and PVT (*p* = 0.20), there was no difference in neuronal activation following saline exposure in the light and dark phase. Representative images for neuronal activation in the PVN following saline or MA exposure in the day and night are shown in **(D)**. *n* = 10 mice/treatment/time period.

Some regions showed only a profound increase in c-Fos positive cells following MA in the day. A significant interaction between treatment and time was found in the PVN [*F*_(1, 36)_ = 26.51, *p* < 0.001; Figure 3A], PVT [*F*_(1, 36)_ = 9.26, *p* < 0.01; Figure 3B], and ARC [*F*_(1, 36)_ = 90.17, *p* < 0.001; Figure 3C]. In the light phase, the number of c-Fos positive cells was higher following MA than saline exposure in the PVN (*p* < 0.001), PVT (*p* < 0.001), and ARC (*p* < 0.001). In the dark phase, the number of c-Fos positive cells was only higher following MA than saline exposure in the PVT (*p* = 0.028) but not in the PVN or ARC. In the PVN, in addition to the MA × time interaction, there were also main effects of MA [*F*_(1, 36)_ = 38.09, *p* < 0.001] and time [*F*_(1, 36)_ = 12.89, *p* < 0.001; Figure 3A). Representative images for neuronal activation in the PVN following saline or MA exposure in the light and dark phases are shown in Figure 3D. In addition to the MA × time interaction, there were also main effects of MA in the PVT [*F*_(1, 36)_ = 50.24, *p* < 0.001; Figure 2B], and ARC [*F*_(1, 36)_ = 15.05, *p* < 0.001; Figure 2C]. As there was a treatment × time interaction in these brain areas, we also assessed whether for each treatment there was a significant difference between neuronal activation in the light and dark phase. In the PVN (*p* < 0.0001), PVT (*p* = 0.01), and ARC (*p* = 0.03) neuronal activation following MA exposure was significantly greater in the light than dark phase. In contrast, in the ARC, neuronal activation following saline exposure was significantly greater in the dark than the light phase (*p* < 0.01). In the PVN (*p* = 0.12) and PVT (*p* = 0.20), there was no difference in neuronal activation following saline exposure in the light and dark phase.

### Pattern 4. brain areas that showed effects of treatment and time

**Figure 4:**
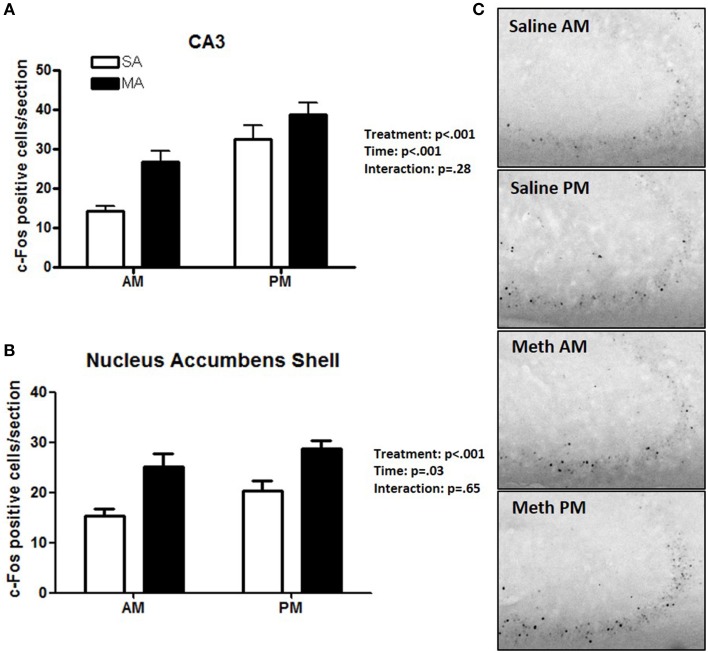
**Brain areas that showed effects of treatment and time**. In the CA3 **(A)** and NACs **(B)**, MA and time affected the number of c-Fos positive cells and no MA × time interaction was seen. Representative images for neuronal activation in the CA3 following saline or MA exposure in the day and night are shown in **(C)**. *n* = 10 mice/treatment/time period.

Two brain regions showed effects of treatment and time but no treatment × time interaction. The CA3 showed main effects of MA [*F*_(1, 36)_ = 38.09, *p* < 0.001] and time [*F*_(1, 36)_ = 30.32, *p* < 0.001] but no MA × time interaction (Figure 4A). The NACs also showed main effects of MA [*F*_(1, 36)_ = 23.28, *p* < 0.001] and time [*F*_(1, 36)_ = 5.20, *p* = 0.028] but no MA × time interaction (Figure 4B). Representative images for neuronal activation in the CA3 following saline or MA exposure in the light and dark phases are shown in Figure 4C.

### Pattern 5. brain areas that showed no treatment or time effects or treatment × time interaction

**Figure 5:**
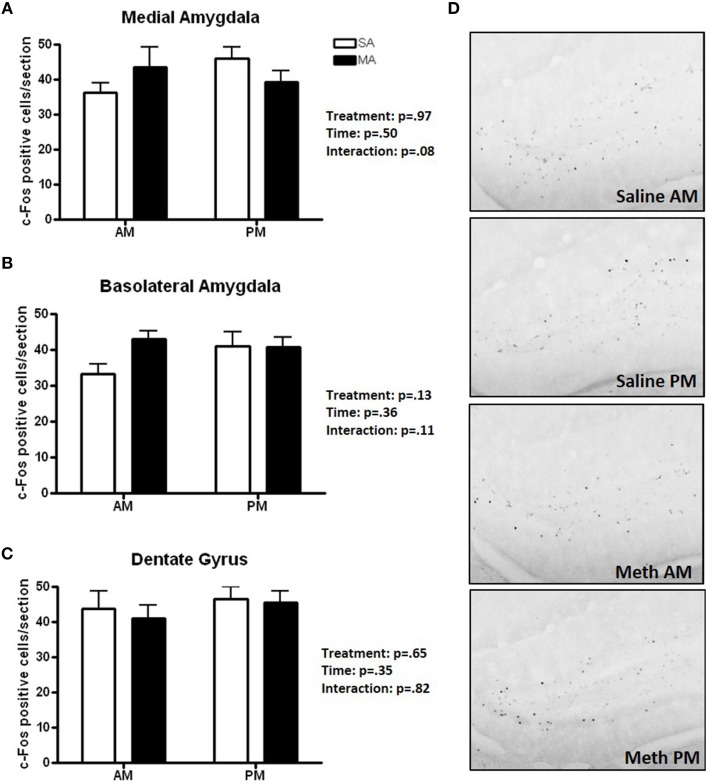
**Brain areas that showed no treatment or time effects or a treatment × time interaction**. There were no significant effects of MA, time, or an interaction between MA and time in the MEA **(A)**, BLA **(B)**, and DG **(C)**. There was a trend toward an interaction in the MEA (*p* = 0.08) but that did not reach significance. Representative images for neuronal activation in the DG following saline or MA exposure in the day and night are shown in **(D)**. *n* = 10 mice/treatment/time period.

Three brain regions showed neither overall effects of MA or time or a MA × time interaction (Figure 5): the MEA (Figure 5A), BLA (Figure 5B), and DG (Figure 5C). There was a trend toward an interaction between MA and time in the MEA (*p* = 0.08) with an increase in the number of c-Fos positive cells following MA exposure in the day but decrease in the number of c-Fos positive cells following MA exposure in the night but that did not reach significance. Representative images for neuronal activation in the DG following saline or MA exposure in the light and dark phases are shown in Figure 5D.

### c-Fos-AVP dual-labeling

**Figure 6:**
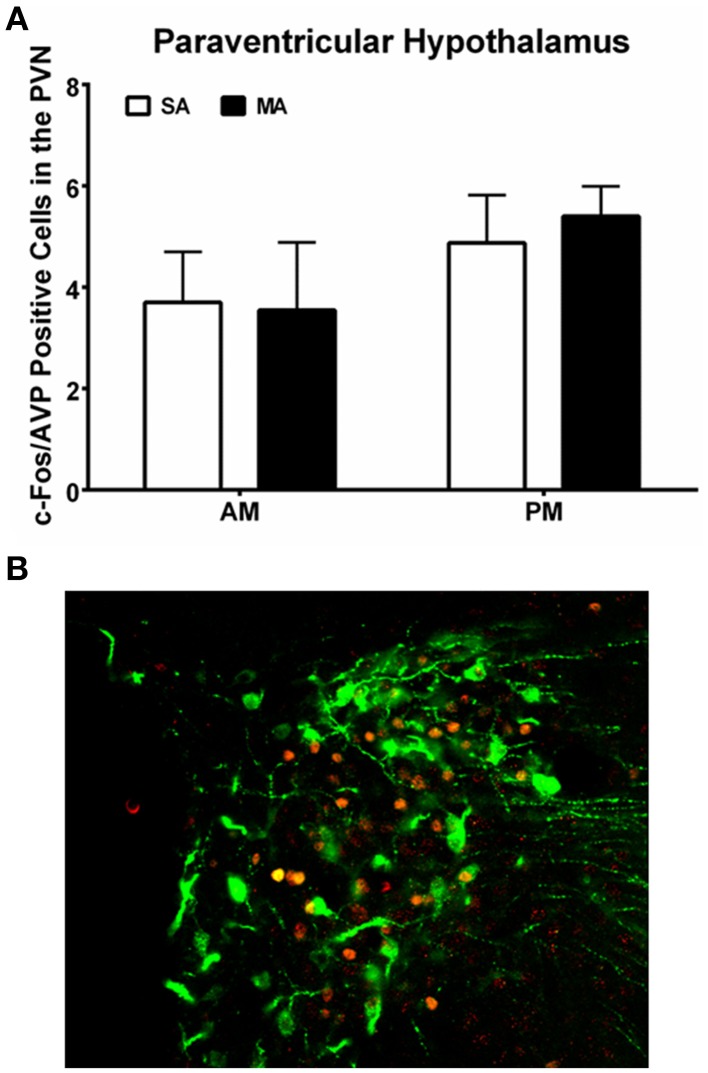
**(A)** Similar c-Fos/AVP dual-labeling in the PVN following MA exposure in the AM and PM. Mice treated with MA in the day and night showed no increase in c-Fos/AVP-ir cells in the PVN compared to saline-treated mice. *n* = 10 mice/treatment/time period. **(B)** Representative panel of c-Fos (red) and AVP (green) from a mouse treated with MA in the AM is shown.

Analysis of the number of c-Fos/AVP dual labeled cells in the PVN revealed comparable numbers in all four experimental groups and no effect of MA treatment (Figure 6A; see Figure 6B for a representative image of c-Fos/AVP double labeling). There was no effect of MA treatment on the number of c-Fos/AVP dual labeled cells in the SCN either [*F*_(1, 12)_ = 0.16, *p* = 0.69, data not shown].

### Functional connectivity analysis

We inferred a measure of functional connectivity between brain regions, following the general methodology introduced by Schiltz et al. (2007). We started by computing the correlations between all distinct brain regions; summing the absolute values of these connectivity values resulted in a measure of functional connectivity of each brain region. It is conceivable that spurious correlations between these quantities could result in false positives, or connectivity values that are due to chance. To guard against this possibility, we employed a randomization procedure. We randomized the order of the samples (*N* = 10, 000) and we recomputed the connectivity values for each region utilizing the randomized data. The connectivity values inferred utilizing the correct sample order were compared against the distribution of these random connectivity values and quantified utilizing *Z*-scores. Significant values (*Z* > 2) were taken to signify connectivity that is unlikely to arise by pure chance. Nearly all of the regions examined, the connectivity values were significantly higher than values derived from randomized data (see Table 2). In particular, VMH appears highly connected following MA exposure but not after saline.

**Table 2:** **Region connectivity *Z*-scores in brains of methamphetamine (MA) and saline (SA) treated mice^a^**.

	**SCN**	**PVN**	**PVT**	**CEA**	**BLA**	**MEA**	**DG**	**CA1**	**CA3**	**ILC**	**ARC**	**VMH**	**DMH**	**BNST**	**CIN**	**NAC core**	**NACshell**
MA	2.59	3.17	1.15	2.23	1.65	1.84	3.17	2.29	2.61	2.67	1.89	**2.65**	3.52	0.21	1.65	1.32	2.37
SA	2.63	1.85	1.15	0.72	3.23	3.58	1.61	3.11	3.55	5.47	4.18	**0.27**	2.06	2.46	2.54	3.33	3.83
*p*	0.50	0.21	0.49	0.14	0.81	0.84	0.18	0.44	0.71	0.95	0.83	**0.008**	0.25	0.94	9.69	0.78	0.81

a *Each region connectivity value is compared to connectivity values from randomized samples. A majority of the regions have Z-scores above 2 and p-values < 0.05 for at least one of the conditions*.

*There is a large difference in connectivity between MA and saline for the VMH (indicated in bold). This difference was subsequently determined to be statistically significant by a bootstrapping procedure as described in the results section*.

**Figure 7:**
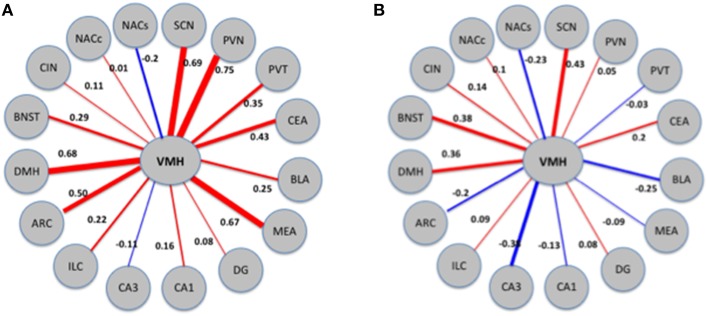
**Patterns of connectivity of the VMH region in MA exposed (A) and MA naïve (B) mice**. The thickness and color of the edges (red for positive and blue for negative) corresponds to the strength and direction (positive or negative) of the correlations. VMH displays changes in correlations with a number of other regions; the combined effects of these changes is quantified as change in connectivity.

Next, we directly tested the hypothesis that VMH connectivity is significantly altered following exposure to MA. As detailed in Methods, we employed a bootstrapping procedure which involved combining the MA and saline data, randomly assigning each sample to one of two mixed groups (*N* = 10, 000), and computing the differences between these groups. Differences between groups arranged in this manner are expected to be small and to reflect spurious changes arising from pure chance. Compared with this distribution of random differences, the difference in VMH connectivity between the MA exposed and saline exposed networks corresponds to a *Z*-score of 2.6 and a bootstrap *p*-value. We illustrate these results in Figure 7.

For a majority of regions examined, the connectivity values were significantly higher than values derived from randomized data (see Table 2).

Next, we inquired whether exposure to MA disrupts the pattern of connectivity between brain regions. The most affected region was the VMH, where the difference in connectivity between the MA exposed and saline exposed networks corresponds to a *Z*-score of 2.6. We illustrate these results in Figure 7.

## Discussion

The results of this study demonstrated that, within the limited number of the brain areas analyzed, there are at least five distinct patterns of neuronal activation (Table 1): (1) brain areas such as the CEA, BNST, CA1, CIN, and NACc that show only an effect of treatment. In the CA1, more neuronal activation following exposure to saline is seen in the dark than light phase (*p* < 0.05) and there was a trend toward such an effect in the ILC (*p* = 0.054). This circadian-dependent effect was not seen in the CEA, BNST, and NACc; (2) brain areas such as the SCN and VMH that showed only an effect of time; (3) brain areas such as the PVN, PVT, and ARC, which show an effect of treatment and a treatment × time interaction with a more profound activation during the light compared to the dark phase; (4) brain areas such as CA3 and NACs which show effects of treatment and time; and (5) brain areas such as MEA, BLA, and DG that show no neuronal activation following MA exposure and comparable neuronal activation in the light and dark phases. We recognize that in brain areas with elevated basal levels of c-Fos no effects might have been seen due to a potential ceiling effect. The pattern of neuronal activation following MA exposure that shows more profound activation during the light compared to the dark phase highlights the importance of time of drug taking (for review, see Hasler et al., 2012).

The difference in connectivity between the MA and saline exposed networks was the greatest in the VMH. The enhanced functional connectivity between the VMH and the PVN, BLA, and MEA following MA exposure is consistent with a stress response (Dunn, 1987; Sánchez et al., 1995; Walker and Davis, 1997; Fendt et al., 2005). Consistent with this notion, MA activates the HPA axis (Williams et al., 2000; Acevedo et al., 2008; Zuloaga et al., 2014) and chronic stress augments the acute and long-term effects of MA (Matuszewich and Yamamoto, 2004). In brain areas like the PVN, the circadian-dependent pattern of activation following MA exposure resembles that seen for stress-induced activation of the HPA axis. The pattern of HPA axis activation shows low baseline stress hormone levels and a stronger stress response in the early light period, compared to a high baseline and reduced stress response in the late light period, close to the onset of the dark period (Raber et al., 2000).

Within the limitations of the current study, AVP does not seem to play a significant role in the MA-induced neuronal activation in the PVN in the light phase. However, we recognize that AVP might play role in the effect of MA at other time points or in other brain areas, such as the VMH in which AVP was shown to provide a diurnal rhythm of stimulatory input in rats (Kalsbeek et al., 1996). Future efforts are warranted to determine the role of AVP in MA-induced circadian-dependent neuronal activation.

When neuronal activation was analyzed in individual brain regions, MA exposure did not affect neuronal activation in the VMH in either the light or dark phase compared to saline exposure, a pattern similar to that seen in the SCN. However, the functional connectivity data with a profound difference in connectivity between the MA exposed and saline exposed networks might suggest a role for VMH in MASCO. Consistent with this hypothesis, the VMH is involved in synchronization of rhythms to periodic feeding (Inouye, 1983) and in circadian rhythms of circulating levels of insulin, glucose, and triglycerides (Kraeuchi et al., 1985; Egawa et al., 1993; Mitsushima et al., 1994). However, we recognize that a brain region involved in MASCO might show a response in neuronal activation following MA exposure in either the light and/or dark phases and that at present too little is known about MASCO to draw any definite conclusion.

The alterations in coordinated activity in brain following MA exposure might play an important role in the detrimental effects of MA. In addition to environmental factors, genetic factors also play an important role in the strengths of network connectivity (Glahn et al., 2010; Fornito et al., 2011) and might play a role in modulating individual susceptibility to effects of MA. Recently, synaptic genes and polymorphisms in these genes were shown to be particularly important in determining functional connectivity in brain and to be associated with resting state measures of functional magnetic resonance imaging (fMRI) and neurological diseases (Richiardi et al., 2015). Future efforts are warranted to determine how the connectivity data revealed using c-Fos immunoreactivty in this study compare to MRI measures following MA exposure.

In summary, the data of this study show distinct patterns of neuronal activation following MA exposure. In several brain areas, neuronal activation following exposure to MA is stronger in the light than the dark phase highlighting the importance of considering circadian periods in defining experimental conditions and understanding the mechanisms underlying detrimental effects of MA exposure to brain function. The enhanced functional connectivity between the VMH and areas like the PVN, BLA and MEA following MA exposure is consistent with a stress response. Future efforts are warranted to determine the role of the VMH in effects of MA on the brain.

## Author contributions

DZ: experimental design; acquisition and analysis of data, interpretation of the data and input to the manuscript. OI: analysis of data, interpretation of the data, and input to the manuscript. SW: acquisition and analysis of data, and input to the manuscript. DE: acquisition and analysis of data, and input to the manuscript. TM: acquisition of data and input to the manuscript. BS: acquisition of data and input to the manuscript. CA: interpretation of the data and input to the manuscript. JR: experimental design, interpretation of the data and input to the manuscript.

### Conflict of interest statement

The authors declare that the research was conducted in the absence of any commercial or financial relationships that could be construed as a potential conflict of interest.

## References

[B1] Abekawa T. Ohmori T. Koyama T. (1996). Effects of nitric oxide synthesis inhibition on methamphetamine-induced dopaminergic and serotonergic neurotoxicity in the rat brain. J. Neural Transm. 103, 671–680. 10.1007/BF01271227 8836929

[B2] Acevedo S. F. Pfankuch T. Van Meer P. Raber J. (2008). Role of histamine in short- and long-term effects of methamphetamine on the developing mouse brain. J. Neurochem. 107, 976–986. 10.1111/j.1471-4159.2008.05673.x 18786166 PMC3172696

[B3] Antoni F. A. (1986). Hypothalamic control of adrenocorticotrophin secretion: advances since the discovery of 41-residue corticotrophin-releasing factor. *Endocr*. Rev. 7, 351–378. 10.1210/edrv-7-4-351 3023041

[B4] Brady A. M. Glick S. D. O'Donnell P. (2003). Changes in electrophysiological properties of nucleus accumbens neurons depend on teh extent of behavioral sensitization to chronic methamphetamine. Ann. N.Y. Acad. Sci. 1003, 358–363. 10.1196/annals.1300.026 14684461

[B5] Broening H. W. Pu C. Vorhees C. V. (1997). Methamphetamine selectively damages dopaminergic innervation to the nucleus accumbens core shile sparing the shell. Synapse 27, 153–160. 9266776 10.1002/(SICI)1098-2396(199710)27:2<153::AID-SYN6>3.0.CO;2-D

[B6] Chang L. Cloak C. Jiang C. S. Farnham S. Tokeshi B. Buchtal S.. (2009). Altered neurometabolites and motor integration in children exposed to methamphetamine in utero. Neuroimage 48, 391–397. 10.1016/j.neuroimage.2009.06.062 19576287 PMC3142567

[B7] Chang L. Ernst T. Speck O. Patel H. DeSilva M. Leonido-Yee M.. (2002). Perfusion MRI and computerized cognitive test abnormalities in abstinent methamphetamine users. Psychiatry Res. 114, 65–79. 10.1016/S0925-4927(02)00004-5 12036507

[B8] Chang L. Smith L. M. LoPresti C. Yonekura M. Kuo J. Walot I.. (2004). Smaller subcortical volumes and cognitive deficits in children with prenatal methamphetamine exposure. Psychiatry Res. 132, 95–106. 10.1016/j.pscychresns.2004.06.004 15598544

[B9] Dobkin C. Nicosia N. (2009). The war on drugs: methamphetamine, public health and crime. Am. Econ. Rev. 99, 324–349. 10.1257/aer.99.1.324 20543969 PMC2883188

[B10] Dornhorst A. Carlson D. E. Seif S. M. Robinson A. G. Zimmerman E. A. Gann D. S. (1981). Control of release of adrenocorticotropin and vasopressin by the supraoptic and paraventricular nuclei. Endocrinology 108, 1420–1424. 10.1210/endo-108-4-1420 6258907

[B11] Dunn J. D. (1987). Plasma corticosterone responses to electrical stimulation of the bed nucleus of the stria terminalis. Brain Res. 407, 327–331. 10.1016/0006-8993(87)91111-5 3567648

[B12] Eastwood E. Allen C. N, Raber, J. (2012). Effects of neonatal methamphetamine and thioperamide exposure on spatial memory retention and circadian activity later in life. Behav. Brain Res. 230, 229–236. 10.1016/j.bbr.2012.02.003 22330947 PMC3310251

[B13] Egawa M. Inoue S. Satoh S. Takamura Y. Nagai K. Nakagawa H. (1993). Acute and chronic effects of VMH lesions on circadian rhythms in food intake and metabolites. Brain Res. Bull. 31, 293–299. 10.1016/0361-9230(93)90220-6 8490728

[B14] Fendt M. Siegl S. Steiniger-Brach B. (2005). Noradrenaline transmission within the ventral bed nucleus of the stria terminalis is critical for fear behavior induced by trimethylthiazoline, a component of fox odor. J. Neurosci. 25, 5998–6004. 10.1523/JNEUROSCI.1028-05.2005 15976089 PMC6724787

[B15] Fornito A. Zalesky A. Bassett D. S. Meunier D. Ellison-Wright I. Yücel M.. (2011). Genetic influences on cost-efficient organization of human cortical functional networks. J. Neurosci. 31, 3261–3270. 10.1523/JNEUROSCI.4858-10.2011 21368038 PMC6623940

[B16] Franklin K. B. J. Paxinos G. (1997). The Mouse Brain in Stereotaxic Coordinates. San Diego, CA: AcademicPress.

[B17] French L. Pavlidis P. (2011). Relationships between gene expression and brain wiring in the adult rodent brain. PLoS Comput. Biol. 7:e1001049. 10.1371/journal.pcbi.1001049 21253556 PMC3017102

[B18] Gill R. Datta S. Datta S. (2014). dna: An R package for differential network analysis. Bioinformation 10, 233–234. 10.6026/97320630010233 24966526 PMC4070055

[B19] Glahn D. C. Winkler A. M. Kochunov P. Almasy L. Duggirala R. Carless M. A.. (2010). Genetic control over the resting brain. Proc. Natl. Acad. Sci. U.S.A. 107, 1223–1228. 10.1073/pnas.0909969107 20133824 PMC2824276

[B20] Hasler B. P. Smith L. J. Cousins J. C. Bootzin R. R. (2012). Circadian rhythms, sleep, and substance abuse. Sleep Med. Rev. 16, 67–81. 10.1016/j.smrv.2011.03.004 21620743 PMC3177010

[B21] Honma K. Honma S. Hiroshige T. (1986). Disorganization of the rat activity rhtythm by chronic treatment with methamphetamine. Physiol. Behav. 38, 687–695. 10.1016/0031-9384(86)90265-9 3823184

[B22] Honma S. Honma K.-I. Hiroshige T. (1991). Methamphetamine effects on rat circadian clock depend on actograph. Physiol. Behav. 49, 787–795. 10.1016/0031-9384(91)90319-J 1881985

[B23] Hosseini S. M. Gat F. (2012). A graph-theoretical analysis toolbox for analyzing between-group differences in large-scale structural and functional brain networks. PLoS ONE 7:e40709. 10.1371/journal.pone.0040709 22808240 PMC3396592

[B24] Inouye S. (1983). Does the ventromedial hypothalamic nucleus contain a self-sustained circadian oscillator associated with periodic feedings? Brain Res. 279, 53–63. 10.1016/0006-8993(83)90162-2 6640356

[B25] Kalsbeek A. van Heerikhuize J. J. Wortel J. Buijs R. M. (1996). A diurnal rhythm of stimulatory input to the hypothalamo-pituitary-adrenal system as revealed by timed intrahypothalamic administration of the vasopressin V1 antagonist. J. Neurosci. 16, 5555–5565. 8757267 10.1523/JNEUROSCI.16-17-05555.1996 PMC6578885

[B26] Koob G. F. Volkov N. D. (2010). Neurocircuitry of addiction. Neuropsychopharmacology 35, 217–238. 10.1038/npp.2009.110 19710631 PMC2805560

[B27] Kraeuchi K. Rudolph K. Wirz-Justice A. Feer A. (1985). Similarities in feeding behavior of chronic methamphetamine treated and withdrawn rats to VMH lesioned rats. Pharmacol. Biochem. Behav. 23, 917–920. 10.1016/0091-3057(85)90092-9 4080776

[B28] Masubuchi S. Honma S. Abe H. Nakamura W. Honma K. (2001). Circadian activity rhtyhm in methamphetamine-treated Clock mutant mice. Eur. J. Neurosci. 14, 1177–1180. 10.1046/j.0953-816x.2001.01749.x 11683910

[B29] Matuszewich L. Yamamoto B. K. (2004). Chronic stress augments the long-term and acute effects of methamphetamine. Neuroscience 124, 637–646. 10.1016/j.neuroscience.2003.12.007 14980734

[B30] Mitsushima D. Yokawa T. Nishihara M. Takahashi M. (1994). Attenuation of the expression of cricadian rhythms by chronic outputs from the VMH in rats. Physiol. Behav. 56, 891–899. 10.1016/0031-9384(94)90320-4 7824588

[B31] Mohawk J. Miranda-Anaya M. Tataroglu O. Menaker M. (2009). Lithium and genetic inhibition of GSK3b enhance the effect of methamphetamine on circadian rhythms in the mouse. Behav. Pharmacol. 20, 174–183. 10.1097/FBP.0b013e32832a8f43 19339873 PMC2893036

[B32] Olsen R. H. Allen C. N. Derkach V. A. Phillips T. J. Bellknap J. K. Raber J. (2013). Impaired memory and reduced sensitivity to the circadian period lengthening effects of methamphetamine in mice selected for high methamphetamine consumption. Behav. Brain Res. 256, 197–204. 10.1016/j.bbr.2013.08.015 23954232 PMC3815974

[B33] Raber J. Akana S. F. Bhatnager S. Dallman M. F. Wong D. Mucke L. (2000). Hypothalamic-pituitary-adrenal dysfunction in *Apoe*^−∕−^ mice: possible role in behavioral and metabolic alterations. J. Neurosci. 20, 2064–2071. Available online at: http://www.jneurosci.org/content/20/5/2064.full.pdf+html 10684907 10.1523/JNEUROSCI.20-05-02064.2000 PMC6772921

[B34] Raber J. Sorg O. Horn T. F. W. Yu N. Koob G. F. Campbell I. L.. (1998). Inflammatory cytokines: putative regulators of neuronal and neuro-endocrine function. Brain Res. Rev. 26, 320–326. 10.1016/S0165-0173(97)00041-6 9651548

[B35] Richiardi J. Altmann A. Milazzo A. Chang C. Chakravary M. Banaschewski T.. (2015). Correlated gene expression supports synchronous activity in brain networks. Science 348, 1241–1244. 10.1126/science.1255905 26068849 PMC4829082

[B36] Sánchez M.M. Aguado F. Sanchez-Toscano F. Saphier D. (1995). Effetcs of prolonged social isolation on responses of neurons in the bed nucleus of the stria terminalis, preoptic area, and hypothalamic paraventricular nucleus to stimulation of the medial amygdala. Psychoneuroendocrinology 20, 525–541. 10.1016/0306-4530(94)00083-M 7675937

[B37] Schiltz C. A. Bremer Q. Z. Landry C. F. Kelley A. E. (2007). Food-asociated cues alter forebrain functional connectivity as assessed with immediate early gene and proenkephalin expression. BMC Biol. 5, 1–20. 10.1186/1741-7007-5-16 17462082 PMC1868707

[B38] Sommers I. Baskin D. Baskin-Sommers A. (2006). Methamphetamine use among young adults: health and social consequences. Addict. Behav. 31, 1469–1476. 10.1016/j.addbeh.2005.10.004 16309848

[B39] Tataroglu O. Davidson A. J. Benvenuto L. J. Menaker M. (2006). The methamphetamine-sensitive circadian oscillator (MASCO) in mice. J. Biol. Rhythms 21, 185–194. 10.1177/0748730406287529 16731658

[B40] Turnbull A. V. Rivier C. (1996). Corticotropin-releasing factor, vasopressin, and prostaglandins mediate, and nitric oxide restrains, the hypothalamic-pituitary-adrenal reponse to acute local inflammation in the rat. Endocrinology 137, 455–463. 8593789 10.1210/endo.137.2.8593789

[B41] Walker D. L. Davis M. (1997). Double dissociation between the involvement of the bed nucleus of the stria terminalis and the central nucleus of the amygdala in startle increases produced by conditioned versus unconditioned fear. J. Neurosci. 17, 9375–9383. 9364083 10.1523/JNEUROSCI.17-23-09375.1997 PMC6573581

[B42] Williams M. T. Inman-Wood S. L. Morford L. L. McCrea A. E. Ruttle A. M. Moran M. S.. (2000). Preweaning treatment with methamphetamine induces increases in both corticosterone and ACTH in rats. Neurotoxicol. Teratol. 22, 751–759. 10.1016/S0892-0362(00)00091-X 11106868

[B43] Wolkoff D. (1997). Methamphetamine abuse: an overview for health care professionals. Hawaii Med. J. 56, 34–36. 9063008

[B44] Wotjak C. T. Kubota M. Liebsch G. Montkowski A. Holsboer F. Neumann I.. (1996). Release of vasopressin within the rat paraventricular nucleus in response to emotional stress: a novel mechanism of regulating adrenocorticotropic hormone secretion. J. Neurosci. 16, 7725–7732. 8922428 10.1523/JNEUROSCI.16-23-07725.1996 PMC6579083

[B45] Uchihashi Y. Kuribara H. Yasuda H. Umezu T. Tadokoro S. (1994). Long-continuous observation of the effects of methamphetamine on wheel-running and drinking in mice. Prog. Neuropsychopharmacol. Biol. Psychiatry 18, 397–407. 10.1016/0278-5846(94)90071-X 8208988

[B46] Zuloaga D. G. Johnson L. A. Agam M. Raber J. (2014). Sex differences in activation of the hypothalamic-pituitary-adrenal axis by methamphetamine. J. Neurochem. 129, 495–508. 10.1111/jnc.12651 24400874 PMC3997586

