# Supplementary material for: Enhanced functional connectivity involving the ventromedial hypothalamus following methamphetamine exposure
Source: Front Neurosci. 2015 Sep 23;9:326. doi: 10.3389/fnins.2015.00326 (PMC4585047; doi:10.3389/fnins.2015.00326)
Supplement: Supplementary file 1 [file DataSheet1.PDF]

```
# read in the R libraries
library(stats)
#library(WGCNA)

setwd("/Users/iancuo/workDir/conn_Ieg")
rawCircMethData=read.csv("data/circadianMethNew.csv")
dataCircMethCounts= rawCircMethData[,5:21]

dataAmMeth=
as.matrix(dataCircMethCounts[rawCircMethData[,"TX"]=="METH" &
rawCircMethData[,"Time"]=="am",])
dataPmMeth=
as.matrix(dataCircMethCounts[rawCircMethData[,"TX"]=="METH" &
rawCircMethData[,"Time"]=="pm",])
dataAmSaline=
as.matrix(dataCircMethCounts[rawCircMethData[,"TX"]=="SALINE" &
rawCircMethData[,"Time"]=="am",])
dataPmSaline=
as.matrix(dataCircMethCounts[rawCircMethData[,"TX"]=="SALINE" &
rawCircMethData[,"Time"]=="pm",])

dataMeth=rbind(dataAmMeth, dataPmMeth)
dataSaline=rbind(dataAmSaline, dataPmSaline)

brainAreas=colnames(dataAmMeth)

########################################################################
###########################################
connMatrixMeth=cor(dataMeth,dataMeth, use="pairwise.complete.obs")
connMatrixSaline=cor(dataSaline,
dataSaline,use="pairwise.complete.obs")

connMatrixMeth=connMatrixMeth-diag(x=diag(connMatrixMeth),
nrow=dim(connMatrixMeth)[1], ncol=dim(connMatrixMeth)[2])
connMatrixSaline=connMatrixSaline-diag(x=diag(connMatrixSaline),
nrow=dim(connMatrixSaline)[1], ncol=dim(connMatrixSaline)[2])

regionConnMeth=rowSums(abs(connMatrixMeth))
regionConnSaline=rowSums(abs(connMatrixSaline))

no.perms=10000
permResultsMeth=array(data=NA,dim=c(no.perms, length(regionConnMeth)),
dimnames=list(1:no.perms,colnames(connMatrixMeth) ) )
permResultsSaline=array(data=NA,dim=c(no.perms,
length(regionConnSaline)),
dimnames=list(1:no.perms,colnames(connMatrixMeth) ) )

for (nperm in 1:no.perms) {
  randomSamples=sample(1:(dim(dataMeth)[1]), (dim(dataMeth)[1]),
replace=T)
```

```
  connMatrixMethPerm=cor(dataMeth,dataMeth[randomSamples,],
use="pairwise.complete.obs")
  connMatrixSalinePerm=cor(dataSaline,
dataSaline[randomSamples,],use="pairwise.complete.obs")

  connMatrixMethPerm=connMatrixMethPerm-
diag(x=diag(connMatrixMethPerm), nrow=dim(connMatrixMethPerm)[1],
ncol=dim(connMatrixMethPerm)[2])
  connMatrixSalinePerm=connMatrixSalinePerm-
diag(x=diag(connMatrixSalinePerm), nrow=dim(connMatrixSalinePerm)[1],
ncol=dim(connMatrixSalinePerm)[2])

  regionConnMethPerm=rowSums(abs(connMatrixMethPerm))
  regionConnSalinePerm=rowSums(abs(connMatrixSalinePerm))

  permResultsMeth[nperm,]=regionConnMethPerm
  permResultsSaline[nperm,]=regionConnSalinePerm

}

zStatsMeth=0* regionConnMeth
zStatsSaline=0* regionConnSaline
empPvalueMeth=zStatsMeth
empPvalueSaline=zStatsMeth

brainAreas=colnames(dataMeth)
for(area1 in brainAreas){
  zStatsMeth[area1]=(regionConnMeth[area1]-
mean(permResultsMeth[,area1]))/sd(permResultsMeth[,area1])
  zStatsSaline[area1]=(regionConnSaline[area1]-
mean(permResultsSaline[,area1]))/sd(permResultsSaline[,area1])

  empPvalueMeth[area1]=sum(regionConnMeth[area1] <
permResultsMeth[,area1])/no.perms
  empPvalueSaline[area1]=sum(regionConnSaline[area1] <
permResultsSaline[,area1])/no.perms

}

table1Updated=round(rbind(zStatsMeth, empPvalueMeth, zStatsSaline,
empPvalueSaline), 3)
write.csv(table1Updated, file="data/table1Updated.csv")
#######################################################################
##########################################
dataTotal=rbind(dataMeth, dataSaline)

permResultsMethVsSaline=array(data=NA,dim=c(no.perms,
length(regionConnMeth)),
dimnames=list(1:no.perms,colnames(connMatrixMeth) ) )
```

```
connMatrixMeth=cor(dataMeth,dataMeth, use="pairwise.complete.obs")
connMatrixSaline=cor(dataSaline,
dataSaline,use="pairwise.complete.obs")

connMatrixMeth=connMatrixMeth-diag(x=diag(connMatrixMeth),
nrow=dim(connMatrixMeth)[1], ncol=dim(connMatrixMeth)[2])
connMatrixSaline=connMatrixSaline-diag(x=diag(connMatrixSaline),
nrow=dim(connMatrixSaline)[1], ncol=dim(connMatrixSaline)[2])

regionConnMeth=rowSums(abs(connMatrixMeth))
regionConnSaline=rowSums(abs(connMatrixSaline))
regionConnDiff=regionConnMeth-regionConnSaline

no.perms=100000
permResultsDiff=array(data=NA,dim=c(no.perms, length(regionConnMeth)),
dimnames=list(1:no.perms,colnames(connMatrixMeth) ) )

for (nperm in 1:no.perms) {
  randomSamplesMeth=sample(1:(dim(dataTotal)[1]), (dim(dataMeth)[1]),
replace=F)
  randomSamplesSaline=sample(1:(dim(dataTotal)[1]), (dim(dataMeth)
[1]), replace=F)

  connMatrixMethPerm=cor(dataTotal[randomSamplesMeth,],
use="pairwise.complete.obs")
  connMatrixSalinePerm=cor(dataTotal[randomSamplesSaline,],
use="pairwise.complete.obs")

  connMatrixMethPerm=connMatrixMethPerm-
diag(x=diag(connMatrixMethPerm), nrow=dim(connMatrixMethPerm)[1],
ncol=dim(connMatrixMethPerm)[2])
  connMatrixSalinePerm=connMatrixSalinePerm-
diag(x=diag(connMatrixSalinePerm), nrow=dim(connMatrixSalinePerm)[1],
ncol=dim(connMatrixSalinePerm)[2])

  regionConnMethPerm=rowSums(abs(connMatrixMethPerm))
  regionConnSalinePerm=rowSums(abs(connMatrixSalinePerm))
  regionConnDiffPerm=regionConnMethPerm-regionConnSalinePerm

  permResultsDiff[nperm,]=regionConnDiffPerm
}

zStatsDiff=0* regionConnMeth
empPvalueDiff=zStatsDiff

for(area1 in brainAreas){
  zStatsDiff[area1]=(regionConnDiff[area1]-
mean(permResultsDiff[,area1]))/sd(permResultsDiff[,area1])
  empPvalueDiff[area1]=sum(regionConnDiff[area1] <
```

```
permResultsDiff[,area1])/no.perms
}


zStatsDiff
empPvalueDiff

table2=round(rbind(zStatsDiff, empPvalueDiff), 3)
write.csv(table2, file="data/table2.csv")
```
